# Congestion

**Published:** 1896-01

**Authors:** 


### EDITORIAL DEPARTMENT.

F. E. DANIEL, M. D., Editor.
S. E. HUDSON, M. D., Managing Editor.
A. J. SMITH. M. D., Galveston, Associate Editor.

EDITORIAL STAFF:

PROF. J. E. THOMPSON, M. D., Texas Medical College, Galveston; Surgery.
PROF. WM. KEILLER, M. D., Texas Medical College, Galveston; Obstetrics and Gynecology.
PROF. DAVID CERNA, M. D., Texas Medical College, Galveston; Therapeutics.
PROF. A. J. SMITH, M. D., Texas Medical College, Galveston; Medicine.
DR. R. H. L. BIBB, Saltillo, Mexico; Foreign Correspondent.

Official organ of the West Texas Medical Association, the Houston District Medical Association, the Austin District Medical Society, the Galveston County Medical Society, and several others.

### CONGESTION.

The term "congestion" called into question by Dr. Collier's letter, published elsewhere in this issue, does not open a new question by any means. It has been under discussion from the early years of the century; and all over the field of medical literature, especially in that of this country, the word is to be encountered as signifying some definite morbific condition, yet clearly employed by the different writers in widely differing senses. Now, it is a "congestive fever," again a "simple congestion" which has been encountered, and in many instances the term evidently has no reference whatever to any especial presence of blood in any particular portion of the body, but is used simply as a synonym for the words malignant or pernicious. The most common employment of the term in the United States, especially in the southern and western parts of the country, is in connection with malarial feves of one or other type; but one hears or sees it used almost as often in connection with a half dozen or more of other processes. A case of influenza may present the symptom, or a case of typhoid fever, or of pneumonia or yellow fever, or of almost any of the severer forms of infection. "Congestion" may prove the immediate cause of death in any instance of cardiac failure from inability of the right heart to perform its labor of driving onward the blood through the lesser circulation, whether in connection with some acute febrile disturbance, or from the gradual lack of compensation of advanced heart disease. An enlarged or cirrhotic liver may interfere with the advance of the blood in the portal system, and "congestion" occur with the gastric and intestinal hemorrhage of greater or less severity. "Congestion," too, is but too frequently used as a cloak for the ignorance of the practitioner who has failed to realize the true import of the symptom, magnifying it into the primary affection causing it, and thus often failing to appreciate the proper application of his remedial measures. Herein lies the gravest objection to its common use, in that it is apt to mislead the attention by its importance as a symptom from the importance of the cause at its origin. Just as the symptom, dropsy, at one time too widely regarded as a disease rather than as a symptom of disease, has come gradually to be appreciated and spoken of in its proper relation, so probably and properly will the appearance of congestion come to be regarded.

Congestion is essentially a state of vascular fullness in one or other part of the body, due to imperfect progression of the blood through the congested vessels from one of a large number of causes. Its common evidence when involving the peripheral vessels, is the bluish appearance of the lips, nails, or even the general surface. It is accompanied by rapid heat dissipation, and hence often by low surface temperature. This last symptom is of course absent in those cases where the congestion appears in the febrile invasion. So, too, it is often accompanied by profuse and clammy perspiration; but this, too, is not apt to be present in the brief congestion of the invasive stage, becoming manifest toward the close of the febrile course. There are thus two periods in the course of fevers when congestion may be present, the period of invasion, and toward the close of the fastigium. In the first, the congestion is usually slight, of brief duration, and of a relatively active type. It is probably the result of a forcing of the blood into the non-resisting capillaries of the surface, and also of the rest of the body, by the usual vascular pressure seen during this stage of an acute course of disease brought about by stimulation, directly or through the nervous apparatus, of the circular muscle fibres of the arteries, and, to a less extent, of the veins, by the various toxins developed by the infectious germs present. As suggested, the congestion of this stage of the disease is not simply of the superficial circulation, but as well of other capillary areas throughout the body. Thus is probably brought about the tendency toward hemorrhage at the beginning of infectious diseases, as in case of the epistaxis of typhoid fever. So, too, as the result of the generalized intracranial pressure from fullness of the great number of small vessels in the cranial vault, in the membranes, and in the brain substance, are to be explained the frequent headaches of this stage of an acute fever. Thus, too, it may be understood why, from distension of the small, thin-walled vessels of the kidney, one should encounter in the period of invasion frequent and free micturitions and the dull pain and sense of weight in the lumbar regions. The distension of the small vessels of the gastric mucous membrane is probably responsible for the nausea and vomiting so often met in this stage. Such a period of arterial and venous constriction (with capillary distension) usually ends in the natural termination of over-stimulation, weakening the relaxation; and this relaxation becomes progressively more marked as, to the paresis of the vascular muscles, is added the degenerative influence of the high temperatures and toxins developed in the course of the case. When this second period of vascular inability is present, the congestion is no longer apt to be uniformly distributed over the surface, is more of a passive type, and hence presents more of a cyanotic appearance than that just mentioned. It is more apt to be seen in the extremities and the dependent portions of the surface; it is also more marked in the central capillary circulations, as of the lungs, kidney or liver. The fault is not from over-pressure; it is twofold, partly because, in the relaxed state of the vessels, there is relatively but a small bulk of blood, and hence under pressure of the heart; and because of the weakened state of the cardiac wall. Because of the relaxed state of the vessels and the progressive degenerative changes in the circulatory muscular system, the low pressure and the attendant tendency to stagnation are apt to be progressive.

The profuse perspirations of the relaxed state now show themselves, especially in the extremities, and the surface with its slowed circulation rapidly cools, so that the apparent temperature is apt to become subnoral. While this relaxation and weakening of the vessel walls go on to marked degrees, hemorrhages are often met, particularly if hæmic changes also exist to render the blood more hydraemic, as in yellow fever and malaria. The slowing of the circulation in the renal area may amount almost to stagnation, and hence, where, in the invasive stage from increased pressure there was poluria, there may now appear suppression, possibly with some degree of hæmaturia. The slowing of the pulmonary circulation permits free exudation through the relaxed and flabby capillary walls, and œdema and hypostatic congestions appear, with their well marked train of symptoms; and death is close at hand. It would seem necessary only to recall these two periods of congestion that they may be easily recognized; as for example, in the stage of rigor and in the sweating stage of the paroxysm of ordinary malarial fever, or in the invasion of typhoid fever and toward the amphibolic stage of the same disease—the first an active congestion, the second a congestion from relaxation.

If, in addition to such trains of circumstances, there be added local obstruction to blood progression in relatively important areas, as in the lungs in pneumonia or other obstruction disease, the general tendency is but augmented and made more apparent; or if to the relaxed state of the circulatory apparatus, there be added previous cardiac weakness from heart disease, the latter but accentuates, and makes more rapid the congestive symptoms, both local and general.

The treatment of the symptom, often so important that it can not be overlooked, then resolves itself into the treatment of the local causes for the prevention of proper blood progression; into the removal of the undue intravascular pressure in the active type, and into the stimulation of the circulation in the relaxed type. Leaving out of consideration, the first of these, as too variable in its different possibilities for the present paper, the second is to be met by the exhibition of relaxant remedies, as the various hydrocarbon antipyretics, veratrum, the antimonials, or by warm baths or blood letting; while the third, the treatment of the relation is to be practiced by stimulation by digitalis, strychnia, alcohol or other similar stimulant remedies.

A. J. S.

---

LET IT be borne in mind that the Texas State Medical Association will meet on the fourth Tuesday in April next, at Fort Worth. Matters of the first importance will be there discussed, and it is highly desirable that every physician who feels a proper pride in his profession, and desires to see it elevated and advanced, and the very name of "medicine" redeemed from the opprobrium cast upon it, should be present. Texas medicine has been, for years, a by-word and a reproach. In Texas anybody can practice medicine who has any kind of a diploma, from anywhere; and many are practicing who have no diploma from anywhere. True, our past failures to attract serious attention to the subject on the part of the legislators, and to secure the legislation which time and again we have endeavored to make them see is absolutely necessary for the public safety, is enough to discourage the most zealous advocate of reform; but the matter must not be left to time or chance. It is a duty which the organized profession of the State owes to the public, that they secure it, and cease not their labors till the ignorant and unscrupulous are driven out of the profession.

At the coming meeting another plan of campaign will be formulated; or else steps will be taken to put into effect the recommendations of the TEXAS MEDICAL JOURNAL on this subject; i. e., to secure co-operation and simultaneous action on the part of all local societies; and have each society to appoint a committee whose duty it shall be to personally see the candidates for the legislature, and make clear to them the necessity of a respectable law with reference to the privilege of practicing medicine; and moreover, to make the vote of the doctors everywhere contingent upon a pledge to not only support such a measure as is needed, but to take an active part in securing its passage. That is the Ohio plan, and there the profession are a unit.

The JOURNAL is pleased to announce that the President, Dr. P. C. Coleman, is taking very active steps, not only in this direction, but by an extensive correspondence he is infusing a renewed interest into the hearts of members, and is doing, in fact, everything possible, to work up a large, enthusiastic and successful meeting. We predict that Dr. Coleman's administration will be signalized by marked success in the advancement of the Association's interests, and from it will date an era of increased prosperity and interest; he will turn the tide, which, for several years, has been adverse, and, as we have often pointed out, at a very low ebb. In this laudable work the JOURNAL bespeaks for its popular and zealous President the aid and co-operation of members in the various sections. Strengthen his hands; talk up the meeting, and do what you can to secure attendance. Fort Worth is central, and easy of access from every part of the State. Low rates will be secured; and then—the Panther City, they say, has grown so fast, that it has pulled up by the roots, every tree growing within forty miles; such a grower is worth going *miles* to see.

Dr. Coleman especially wishes that those gentlemen who have been honored with chairmanships shall show some appreciation of the honor. It is no empty compliment, but each chairman was chosen and elected by the nominating committee because he was considered a representative man of his section who could rally to his support a large contingent. Let these chairmen stir themselves to get up papers for their sections; and at as early date as possible send the name of the subject chosen, to the general secretary, so as to enable him to arrange the programme in time, and intelligently; thus insuring a reading for each paper.

The JOURNAL hopes to see some action taken at this meeting looking to a memorial to the legislature to do something for the epileptics of the State. Surely, unless the doctors direct attention to it, the legislators will hardly move of their own accord; they will know nothing of the subject nor of the necessity for action.

Of the making of medical journals, in Texas at least, there seems to be no end. We have received Vol. 1, No. 1 of the "*The Southwestern Medical and Surgical Reporter*, a journal of medicine and surgery" (December, 1895), published at Fort Worth. Drs. T. J. Bell and F. G. Kirkscey, of Tyler, and Dr. E. D. Capps, of Fort Worth are editors. Notwithstanding that it is "Vol. 1, No. 1," we infer that it is Dr. Bell's "Hygiea," under another name, as, in the salutatory the editors say, "we have changed our name but not our politics or religion." We were not before aware that either politics or religion entered into the make up of the Hygiea; however, the doctor may have been talking through his hat. At any rate, we are sorry to see the Hygiea, whose launching, with a flourish of trumpets and a flashing of poetry, was only last April announced, lower her flag and strike her colors. What's the matter with "sanitary" in Texas? No "sanitary" or "health" journal seems to prosper. The *Texas Sanitarian*, which had a good start, and three years experience, changed its name to the "*Texas Medical News*." Do these gentlemen who start "sanitary" journals (all to fill a long felt want), all envy the "Red Back's" success,—think there is more money and glory in "medical" than in "sanitary?" I know what's the matter with them; it gives them the heart burn to read of how the JOURNAL receives Dr. Merriman when he drops in; of how he touches the button and helps himself to our two-bit havanas and our sherry cobblers; they envy our wealth. ("They'll be sorry" when they see our new $6 overcoat.)

We are not envious. There's room enough for all of us, and we cordially wish our friends success in their new undertaking.

Anent starting medical journals the following from the N. Y. *Medical Record* will be read with interest:

"BOGUS MEDICAL JOURNALISM.—While the *Medical Record* hails with satisfaction the advent of any new medical journal which appears to have a legitimate sphere, and always extends to it such encouragement as lies in its way, it seems right that a word of condemnation should be uttered respecting the too numerous periodicals which are launched upon the profession without any reasonable necessity or justification. Not a few are conceived solely by advertising agents—men who have neither interest nor sympathy with the profession save as they can make it a means of livelihood for themselves—and published at more or less nominal prices in anticipation of a circulation which will enable them to obtain the advertisements upon which they depend for their profits.

"It is unfortunate that there are always medical gentlemen to be found who, for a small sum, are willing to "run" these journals. Again, it not infrequently happens that a medical journal is started by some doctor who is desirous of exploiting his own abilities and skill, and at the same time add to his library such books as he may be able to induce publishers to send to it for "review." We know of journals published in this country, by individuals and associations of physicians, whose almost sole *raison d'être* is the exchanges and editorial copies of books which they obtain, without which they would cease to exist, and to obtain which they must always speak of them in terms of commendation. The publishers, not the subscribers, must be considered first. Medical journals of their class are a delusion and a snare to the profession, and an injury to all rightly conceived and conducted periodicals.

"As we have often stated, we believe in local medical journals and their generous support by their legitimate constituency; and we can not but regret that they should have to compete with journals such as we have mentioned. Of course, there is no way to prevent the publication of these illegitimate journals, and the only recourse in the hands of the profession is, as far as possible, to patronize only those of whose origin and management they have some personal knowledge. In other words, avoid subscribing to medical journals which have not good and well-known pedigrees on the sides of both publisher and editor."

